# Synaptic GluN2A-Containing NMDA Receptors: From Physiology to Pathological Synaptic Plasticity

**DOI:** 10.3390/ijms21041538

**Published:** 2020-02-24

**Authors:** Luca Franchini, Nicolò Carrano, Monica Di Luca, Fabrizio Gardoni

**Affiliations:** Department of Pharmacological and Biomolecular Sciences, University of Milan, Via Balzaretti 9, 20133 Milan, Italy; luca.franchini@unimi.it (L.F.); nicolo.carrano@unimi.it (N.C.); monica.diluca@unimi.it (M.D.L.)

**Keywords:** Glutamate, NMDA receptors, dendritic spines, synaptic plasticity, brain disorders

## Abstract

N-Methyl-d-Aspartate Receptors (NMDARs) are ionotropic glutamate-gated receptors. NMDARs are tetramers composed by several homologous subunits of GluN1-, GluN2-, or GluN3-type, leading to the existence in the central nervous system of a high variety of receptor subtypes with different pharmacological and signaling properties. NMDAR subunit composition is strictly regulated during development and by activity-dependent synaptic plasticity. Given the differences between GluN2 regulatory subunits of NMDAR in several functions, here we will focus on the synaptic pool of NMDARs containing the GluN2A subunit, addressing its role in both physiology and pathological synaptic plasticity as well as the contribution in these events of different types of GluN2A-interacting proteins.

## 1. Introduction

N-Methyl-d-Aspartate Receptors (NMDARs) are ligand- and voltage-gated ionotropic glutamate receptors (iGluRs) promoting Ca^2+^ and Na^+^ influx. In particular, the NMDAR pore channel is blocked by Mg^2+^ at resting potential and this event is removed by an adequate depolarization. Therefore, in the canonical view, activation of NMDARs represents a coincidence detector of presynaptic glutamate release and postsynaptic depolarization [1,2]. This view requires NMDARs be located postsynaptically [3]. Nonetheless, the presence of NMDARs in the presynapse has long been described in several brain areas, like cortex [4,5], hippocampus [6,7], and cerebellum [8,9], and also in the spinal cord [10]. Moreover, in parallel with the classic view of ion influx as the only NMDAR signaling, several studies pointed out a NMDAR metabotropic cascade independently of ion-flow. In particular, the NMDAR metabotropic activity results from a complex intracellular “signalosome”, which is mostly involved in synaptic receptor endocytosis and trafficking [11,12,13,14], and depression of neurotransmission such as NMDAR-dependent Long-Term Depression (LTD) [15,16,17,18,19]. 

NMDARs are tetramers composed by two obligatory GluN1 subunits, associated with two regulatory subunits of GluN2-type and GluN3-type, expressed in several different isoforms (GluN2A-D and GluN3A-B) at different brain regions and at different developmental periods. According to the neurodevelopmental switch theory, GluN2B/GluN2D and GluN3A/GluN3B are more abundant in early developmental stages, whereas GluN2A/GluN2C are more expressed in mature ones [20,21]. 

GluN2A and GluN2B are by far the most abundant NMDAR regulatory subunits expressed in the mammalian brain [20,21,22]. GluN2A-containing NMDARs are highly expressed in the adult hippocampus and neocortex while in other brain areas such as the striatum the GluN2B-containing NMDARs are predominant. Notably, GluN2A-containing NMDARs are more localized at synaptic sites and enriched in the postsynaptic density (PSD) compared to extrasynaptic sites [22,23,24] and display a slower mobility compared to other GluN2-containing ones [25]. 

GluN2A confers unique channel properties to NMDARs containing this subunit, starting from high sensitivity to Mg^2+^ [26]. Chen et al. reported that GluN2A-containing NMDAR mediate large currents, as suggested by the peak current densities [27]. Moreover, this subunit grants the NMDAR a high channel open probability [27]. In postnatal cortical neurons, regardless their age, the presence of GluN2A mRNA was associated to faster NMDAR EPSC compared to negative cells [28]. GluN2 subunits are also the main regulators of the open/close state of the NMDAR. In this context, GluN2A-containing receptors have a reversible calcium-dependent inactivation, whereas other kinds of NMDARs, like the GluN2B-containing ones, do not significantly suffer for calcium-dependent inactivation [29]. Another important feature owned by GluN2A-containing NMDARs is the fast recovery from desensitization [30]. To summarize, GluN2A confers to NMDAR specific electrophysiological properties, like high channel opening probability and conductance, more predisposed to desensitize but with a faster recovery, making these receptors versatile modulators of synaptic activity. 

Synaptic NMDARs mainly mediate pro-survival and synaptic plasticity pathways, whereas extrasynaptic NMDARs are mostly responsible for glutamate excitotoxicity and are detrimental for neuronal functions [23,31]. Furthermore, the balance in synaptic GluN2-type subunits is responsible for adequate glutamatergic neurotransmission, which is altered in several neurological disorders and which is linked to the pathophysiology of brain diseases [22,32,33]. Therefore, understanding the molecular mechanisms regulating NMDAR subunit composition at synapses, such as membrane insertion, and assembly and removal of specific regulatory subunits, represents a pharmacological challenge for the setting up of new therapeutic strategies for neurological disorders where NMDARs play a pivotal role [34,35]. In this review, we will focus on synaptic GluN2A-containing NMDARs, their role in synaptic plasticity and their contribution to pathological plasticity as observed in several brain disorders. 

## 2. NMDAR Structure: Focus on the GluN2A Subunit

All NMDAR subunits contain an extracellular N-terminal domain (NTD), a transmembrane domain (TD), and an intracellular C-terminal domain (CTD). Within NTDs are located the agonist binding site (ABD) for glutamate (on GluN2 subunits) and glycine (on GluN1 and GluN3 subunits) [36]. Zn^2+^ ions are able to inhibit NMDAR currents probably by interacting with pore channel amino acids [37,38]; however, Zn^2+^ can also inhibit specifically GluN2A-containing NMDARs due to an interaction site located on GluN2A NTD. In particular, Zn^2+^ can relate allosterically with four residues (H42, H128, K233, and E266) [39] belonging to GluN2A NTD, therefore providing inhibition and reducing NMDAR currents [40,41,42,43]. GluN2A NTD is also responsible for receptor structural changes in a closed conformation due to extracellular protonation (i.e., pH variations), which were shown to be independent of plasma membrane potential [44,45,46] and agonist binding [47]. This event was shown to be synergistically promoted by other NMDAR inhibitors, such as Zn^2+^ [48]. Recently, R370W and P79R mutations of the *GRIN2A* (Glutamate Ionotropic Receptor NMDA Type Subunit 2A) gene, encoding for the GluN2A subunit, have been associated with augmented and reduced sensitivity to Zn^2+^, respectively [49]. Finally, the NTD of the GluN2A subunit shows an endoplasmic reticulum (ER) retention signal, which is absent on GluN2B homolog [50] probably accounting for different delivery mechanisms to the synapse. 

The TD displays an M2 loop involved in pore channel formation and responsible for Mg^2+^ interaction. In particular, two asparagine residues from M2 loops belonging to the GluN2 and the GluN1 subunits are responsible of the Mg^2+^ blockade [51]. The *C1845A* mutation on *GRIN2A* gene encodes for GluN2AN651K variant at the M2 loop leading to low Mg^2+^ blockade [51], decrease in Ca^2+^ permeability both for di-heteromeric and tri-heteromeric NMDARs, and to epileptic encephalopathy with cognitive impairment [52,53]. 

The long CTD of GluN2 subunits displays the lowest homology among GluN2 isoforms, as well as various length and binding partners [25,32,54]. GluN2 CTDs are necessary for receptor surface dynamics and activation of specific metabotropic signaling [32,55]. Protein–protein interactions at the GluN2A CTD are involved in the lower mobility of GluN2A-containing NMDARs at synapses compared to GluN2B-containing ones [25] (see below) [55]. GluN2 CTD as well as NTD were reported to affect receptor trafficking from the ER, indicating a complex modulation of the receptor delivery to target regions [56]. 

NMDAR can be modulated through subunit phosphorylation of different residues at the CTD, which represent also a well-demonstrated fast mechanism for activation or maintenance of synaptic plasticity events [57]. In particular, CTD phosphorylation is important for the endocytosis/removal of NMDARs, which occurs during synaptic plasticity events [58] and agonist binding [11,12,13,59]. Vissel and collaborators (2001) reported a use-dependent dephosphorylation of NMDAR associated with low amplitude recordings given by augmented endocytosis of the receptors mediated by AP2 and dynamin [11]. This event was dependent on agonist concentrations and time interval of treatments. Furthermore, they identified the GluN2A CTD portions responsible for the endocytosis, such as Tyr842 which is well known to interact with a consensus motif (µ2) on AP2, and additional residues between the 874-1464 fragment [11].

Selective GluN2 antagonists represent pharmacological tools to investigate GluN2 subunits role both in physiological and pathological conditions. As for the GluN2A subunit, the mostly used selective antagonist is NVP-AAM077, which interacts with ABD of GluN2A and Glu781 on GluN1 subunit [60]. The selectivity of NVP-AAM077 for GluN2A over GluN2B is, however, only approximately fivefold, suggesting a tight range of concentrations to be used [61]. This probably explains why the variability of the results obtained with NVP-AAM077 in different experimental systems, thus suggesting that this compound should be used cautiously to investigate GluN2A contribution to neuronal functions.

Researchers recently developed new promising compounds with different pharmacological profile, such as GluN2A Negative Allosteric Modulators (NAMs) and Positive Allosteric Modulators (PAMs). As for GluN2A-NAMs, the most studied is TCN-201, which interacts with the ABD lower portion called S2 of GluN2A and GluN1; in particular, Leu780 and Val783 on GluN2A, whereas Arg755 on GluN1 [62,63,64]. As for GluN2A-PAMs, scientists are making efforts in obtaining clean molecular profiles due to a PAM binding site shared between NMDARs and AMPA receptors (AMPARs) [54]. Among PAMs, GNE-0723 was the first to be identified with a high potency and brain penetrance but poor pharmacokinetic profile [65,66]. After modification of GNE-0723 structure into GNE-5729, almost fivefold increased selectivity against AMPAR was achieved, maintaining good GluN2A potency and specificity [66]. Overall, even if several advances have been performed in the last decade trying to identify novel compounds that are able to modulate selectively the GluN2A-containing NMDARs, additional work is needed to reach this goal.

## 3. GluN2A Subunit: Binding Partners

The GluN2A CTD binds a variety of synaptic proteins with different cellular functions. These protein–protein interactions play a pivotal role in the modulation of numerous properties of GluN2A-containing NMDARs, ranging from the regulation of downstream intracellular signaling to mechanisms involved in the synaptic retention of the receptor.

**Figure 1:**
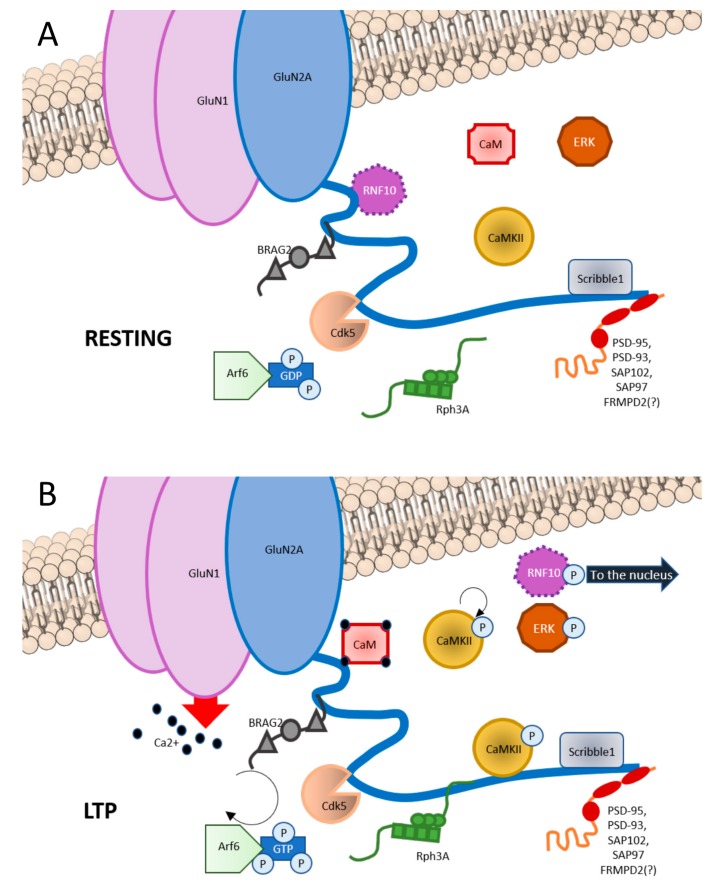
Graphic representation of GluN2A interactors at C-terminal domain (CTD). (**A**) In basal condition, GluN2A CTD binds RNF10 at aa991-1049; BRAG2 at aa1078-1117; Cdk5 at aa1218-1246; CaMKII at aa1389-1464; Scribble1 at 1458-1464; and PSD scaffolding proteins such as PSD-93, PSD-95, SAP102, and SAP97 at aa1461-1464. (**B**) Upon LTP induction, phosphorylation of RNF10 triggers RNF10 detachment and its nuclear translocation. Simultaneously, CaM binds to aa875-1029 of GluN2A. BRAG2 exchanges GDP with GTP to Arf6-GTPase, which is involved is vesicles recycle at the postsynapse, also with Scribble1. Rph3A interaction with GluN2A at aa1349-1389 increases. CaMKII is disinhibited and is recruited at the postsynapse. Ras-ERK downstream cascade is initiated.

The GluN2A CTD domains responsible for the interaction with these partners sometimes overlap thus leading to the competition of different types of proteins for the interaction with the GluN2A subunit (see below). Importantly, it is known that some of these interactions depend on the activation state of the synapse. In particular, interactions at the GluN2A CTD are very often not static but dynamically regulated by synaptic activity and plasticity (Figure 1). Accordingly, different pools of GluN2A-containing NMDAR can be associated to different signalosome. Moreover, several protein–protein interactions described below have been shown to play a key role for Long-Term Potentiation (LTP) induction. Therefore, the knowledge of the interactors at the GluN2A-CTD and their binding mechanisms represent a helpful tool in understanding how GluN2A-containing NMDAR function is regulated.

Among GluN2A CTD interacting partners there are (1) scaffolding proteins, (2) synapse-to-nucleus messengers, (3) protein kinases, and (4) other proteins (see Figure 1). 

### 3.1. Scaffolding Proteins

Several types of scaffolding proteins bind the distal domain of GluN2A CTD and these interactions mainly regulate GluN2A stability at synapses. Members of membrane-associated guanylate kinase (MAGUK) from the Discs Large subfamily (Dlg) such as PSD-95, PSD-93, SAP102, and SAP97 bind to the last three aa (1461–1464) of the GluN2A subunit and are responsible for GluN2A-containing NMDARs retention in PSD [67,68,69,70]. Similarly, the rab-effector protein Rabphilin3A (Rph3A) binds to GluN2A1349-1389 domain at synapses and promotes stabilization of GluN2A-containing NMDARs at the PSD, also thanks to the formation of a triple complex with PSD-95. Interestingly, the formation of this protein complex is triggered by LTP and disruption of Rph3A/GluN2A interaction through cell permeable peptides or Rph3A shRNA rapidly reduces synaptic levels of GluN2A-containing NMDARs and prevents LTP induction [71]. FRMPD2, a scaffolding protein typically present in polarized cells and neurons, was found to interact selectively with GluN2A subunit of NMDARs through its PDZ2. Again, this association is necessary for receptor stabilization in PSD and its disruption leads to reduced GluN2A synaptic levels in CA1 region of hippocampus [72]. Scribble1, a scaffolding protein involved in neural tube closure and presynaptic architecture was also described as a GluN2A binding partner at 1458–1464 domain promoting insertion at the PSD of GluN2A-containing NMDARs via interplay with AP2 [73].

### 3.2. Synapse-to-Nucleus Messengers 

Long-distance NMDAR signaling to the nucleus contributes to activity-dependent regulation of gene expression that feeds back to synaptic function in health and disease. In addition to calcium signals, which represent a major route for communication of NMDAR activity to the nucleus, synaptonuclear protein messengers connect synapses and nucleus enabling bidirectional transfer of information. In particular, several synaptonuclear messengers are associated to NMDAR complex at the glutamatergic postsynaptic compartment and play a key role in the modulation of the LTP [74,75]. Ring Finger protein 10 (RNF10) binds the 991–1049 fragment of GluN2A subunit and migrates to the nucleus through the transporter importin-α after induction of LTP or synaptic NMDAR activation [76].

AIDA-1d was shown to be present in the PSD during resting conditions, and to migrate to the nucleus upon NMDAR activation where it regulates protein synthesis and nucleoli number [77]. An interaction site on GluN2 CTD has not been reported yet for AIDA-1d and it shows to preferentially colocalize and coimmunoprecipitate with GluN2B subunits over GluN2A, suggesting a promiscuous synapse-to-nucleus messenger. However, we cannot exclude AIDA-1d as potentially part of GluN2A-containing NMDAR signaling components. Nevertheless, it seems to mainly affect GluN2B-containing NMDARs assembly and trafficking to the synapse from the endoplasmic reticulum [77].

Extracellular regulated kinase (ERK) is involved in Mitogen-Activated Protein Kinase (MAPK) downstream signaling and a synapse-to-nucleus communication, which still needs to be clarified. Both GluN2A- and GluN2B-containing NMDAR can be found upstream of ERK activation, suggesting ERK as a promiscuous signaling mediator [55]. ERK was shown to be activated by only a subset of dendritic spines modulating CREB and Elk-1 activity [78]. However, there is no direct demonstration of ERK translocation to the nucleus as well as no direct interaction reported yet on GluN2 CTDs. 

### 3.3. Protein Kinases

Several protein kinases play a relevant role at GluN2A-containing NMDAR complexes not only phosphorylating the GluN2A subunit, but also forming protein–protein interactions. Ca^2+^/calmodulin-dependent protein Kinase II (CaMKII) interacts with 1389–1464 domain of GluN2A [79,80] and with different CTD portions of GluN2B subunit (839–1120/1120–1489) depending on the kinase phosphorylation state [81,82]. CaMKII is the most abundant protein at the glutamatergic PSD and is activated by Ca^2+^-Calmodulin complexes driven by NMDAR opening. When this occurs, CaMKII autophosphorylates its autoinhibitory domain in T286, becoming active. The active conformation of CaMKII remains, even when the Ca^2+^ stimulus has subsided, therefore it is also named as “molecular memory” [83,84,85]. The interaction between GluN2A and this kinase was shown to be in an unphosphorylated state, but it is promoted by autophosphorylation of CaMKII [86]. Increased formation of GluN2A/CaMKII complex induces also a disruption of GluN2A/PSD-95 interaction thus leading to a dynamic modification of GluN2A-interacting proteins following NMDAR activation [79]. 

Cyclin-Dependent Kinase 5 (CdK5) binds GluN2A at fragment 1218–1246, regulating receptor recycle and degradation via coordination with the protein AP2 [87,88]. Of relevance, Cdk5-dependent phosphorylation at Ser1232 seems to be important for LTP maintenance in CA1 region of hippocampus [88].

Phospatidyl-inositol-3 kinase (PI3K) was reported to bind GluN2A- or GluN2B-containing NMDAR by interacting with GluN1 CTD. Activation of GluN2A containing NMDAR was shown to trigger PI3K signaling [89,90].

### 3.4. Other Proteins

Guanine nucleotide exchange factors (GEFs): Brefeldin A-resistant Arf guanine nucleotide exchange factor 2 (BRAG2) binds 1078–1117 fragment of GluN2A CTD and mediates exchange of GDP to GTP at ADP-rybosilation factors (Arf) [91]. However, also BRAG1 is able to interact with synaptic GluN2A-containing NMDAR complex, as reported previously by Sakagami [92].

Ras-guanine nucleotide-releasing factor 2 (Ras-GRF2) is a Ca^2+^/Calmodulin (CaM) sensor mediating GluN2A-containing NMDAR signaling in mature neurons [93], in fact its expression is developmentally regulated [94]. In particular, Ras-GRF2 displays a GEF domain, a C-terminal cell division cycle 25 (CDC25) domain for Ras/ERK activation [95,96], and an N-terminal dbl homology (DH) domain activating Rac/p38 pathway [97,98]. Ras-GEF are important mediators of NMDARs late phase signaling, since they involve MAP kinases, ERK1/2 and also CREB phosphorylation which is important for gene expression [94]. However, the domain of interaction between Ras-GRF2 and GluN2A has not been investigated.

IQGAP1 serves as a scaffold protein for different signaling pathways involving B-Raf, Rac1, Lis1, Cdc42, ERK, and MEK. IQGAP1 was found to preferentially interact with GluN2A-PSD-95 complexes compared to GluN2B-containing ones, thus indicating a possible signaling interplay through IQGAP1 between different pools of NMDAR [99,100,101,102].

Calmodulin (CaM) interacts with CTD of GluN2A subunit in a calcium-dependent manner at the 875-1029 domain, through interaction with a tryptophan residue (1014), which is critical for protein–protein interaction [103]. Ca^2+^/Calmodulin complexes can be also considered as a synapse-to-nucleus messengers of the γCaMKII and γCaMKI signaling, regulating gene expression and cognitive functions [104,105,106].

Bip, a recently discovered ER chaperone protein, is involved in the selective delivery of GluN2A-containing NMDAR at the synapse from the dendritic ER [107]. 

## 4. GluN2A Subunit and Synaptic Plasticity

The influence of the NMDAR subunit composition on synaptic plasticity has been subject of a vast number of studies, trying to explain the unsolved question on how different patterns of neuronal stimulation lead to opposite modulation of synaptic strength acting on the same type of receptor. It is commonly accepted that the different levels of calcium influx associated with different degrees of NMDAR activation polarize the direction of plasticity towards LTP or LTD, as they couple to different intracellular signaling pathways. However, it was proposed that distinct subpopulations of NMDARs could exert a finer level of regulation on synaptic potentiation or depression triggered by different activity patterns. In this regard, GluN2A subunit has been intensively studied, as GluN2A-containing NMDAR has particular channel properties that generate distinct calcium dynamics in the postsynapse. Moreover, as described above, GluN2A CTD allows unique intracellular molecular associations with proteins like kinases and phosphatases and synaptonuclear messengers that intrinsically direct the plasticity signaling [33,75,108]. Recently it was also reported a specific metabotropic function of GluN2A-containing NMDAR in mediating glycine-induced potentiation of AMPAR currents through the activation ERK1/2 signaling [109]. The synaptic abundance and motility of this subunit affects the basal state of the spine and provides another level of control on the possibility to induce synaptic strengthening or weakening. In particular, the CA3-CA1 synapse of the hippocampal neurocircuitry represents the most commonly used model for the study of the putative role of different GluN2 subunits in synaptic plasticity. Pharmacological and genetic manipulations, or the combination of the two approaches, have been used in combination with several plasticity protocols. 

### 4.1. Role of GluN2A in LTP

In 2004, a couple of seminal ex vivo studies showed that the selective pharmacological inhibition of GluN2A by NVP-AAM077 blocks tetanus- and pairing-induced LTP in three- to four-week rat hippocampal neurons [110] and HFS-induced LTP in layer II and III cortical neurons [111], but not LTD. A few years later the findings were confirmed on hippocampal neurons using the same antagonist and age of rats [112] and also in two-week-old mice [113]. In vivo pharmacological studies also showed that both intrahippocampal infusion and intraperitoneal injection of NVP-AAM077 was able to abolish LTP induction [114]. The data in these studies seem to point out that GluN2A is a necessary element for LTP. However, the NVP-AAM077 concentration used in the aforementioned studies is relatively high and, as NVP-AAM077 affinity is just 5 times higher to GluN2A than GluN2B, it cannot be excluded that the block of LTP seen is uniquely GluN2A dependent. Moreover, this elevated concentration of NVP-AAM077 was able to induce a drastic reduction of the total NMDAR-mediated currents [110,112] that might have prevented the calcium influx necessary for LTP induction. Given that, the block of LTP induction seemed more likely to have arisen from a threshold effect on the total NMDAR-mediated currents than on the selective inhibition of GluN2A. In support of this theory, the group of Köhr found that in mice treated with a concentration of the antagonist that was titrated not to cause an excessive NMDAR-current reduction, pairing-induced LTP was not impaired [115]. In another study, the same low NVP-AAM077 concentration also did not abolish LTP induction by a theta burst protocol [116], but partially impaired tetanus-induced LTP [117]. Overall, pharmacological studies did not clarify the role of the GluN2A subunits in LTP, even if the data of these studies suggest that GluN2A might have a more relevant function in tetanus-induced LTP, since it resulted the most sensitive to NVP-AAM077 treatment.

Several lines of evidence from a genetic manipulation approach further supported the idea of an involvement of GluN2A in LTP, as in both mice with reduced synaptic GluN2A [118] or no GluN2A [119], LTP is altered, but can be restored though a multiple tetanic induction protocol [120]. A possible explanation for this functional recovery is that a stronger neuronal stimulation can restore the calcium influx that is usually supplied by GluN2A-containing NMDAR in high-frequency stimulation conditions. However, other studies put forward the idea that also GluN2A-interaction with molecular partners is important for synaptic strengthening. It has been shown that form of LTP dependent on the Ras-GRF2/Erk Map Kinase pathway can be sustained by GluN2A subunit-containing NMDARs alone [93], suggesting that unique interactions may occur. This idea seems to be confirmed by another study, in which a GluN2A C-terminal truncation mouse model showed impaired tetanus-induced LTP [121]. However, these mice have a reduction of the total levels of GluN2A, so it is possible that the LTP impairment is caused by the reduction in calcium influx (threshold effect) rather than the loss of the signaling downstream GluN2A. Also, in this case [121], and in another study [122], using more intense stimulation protocols overcomes the LTP induction deficit, probably thanks to the expected increase of calcium influx in the spine. To conclude, these experimental data indicate an involvement of GluN2A in LTP but there is no clear indication for a privileged or necessary role of this subunit in the process induction.

GluN2A has a bidirectional involvement in synaptic plasticity: it regulates and is regulated by it. In particular, LTP is a potent trigger of GluN2A upregulation, especially at synaptic levels. Several evidences seem to indicate that GluN2A rise in the postsynapse is associated to an early mobilization of NMDARs localized in non-synaptic pools and a later increase of total level of the protein. In adult rats HFS-induced LTP is coupled to waves of GluN2A increase in DG total homogenates: a rapid surge 20 min after LTP induction, then the subunit levels go back to the base levels 1h and 4h after LTP to go up again 24h after induction [43]. LTP induced by HFS is also able to promote a GluN2B- to GluN2A-mediated current switch in newborn mice hippocampal slices, in a very short time window [123]. Accordingly, LTP induction increased GluN2A synaptic levels in both hippocampal organotypic cultures from neonatal rats [12] and in synaptosomal fractions of hippocampal slices from 6- to 8-week-old rats [124]. Similarly, Franchini et al. found also in primary hippocampal cultures a synaptic increase of GluN2A driven by chemically induced LTP protocol, with a concurrent increase of its CTD-binding partner Rph3A (see Figure 1). Interestingly, interfering with GluN2A/Rph3A interaction prevents the synaptic increase of GluN2A levels [125].

Baez and coworkers found that only after the establishment of an actual long-term potentiation (after 70 min), but not after 30 min, GluN2A levels were increased [126]. Even if these data seem to contrast with the previously described ones, the different results obtained may arise from the different model used (young adult rats) and the different cellular fraction analyzed (hippocampal homogenates and not synaptic fractions). In accordance, GluN2A mRNA dendritic levels and translation increased 30 min after NMDA stimulation [127], whereas LTP induction immediately increases local translation and membrane insertion of GluN2A and for the 30 min after the induction [128]. 

In conclusion, GluN2A seems to respond to the appropriate stimulus with a fast lateral mobilization from extra-synaptic to synaptic sites and, to sustain synaptic potentiation, with an increase of the local translation and surface expression at longer time points.

### 4.2. Role of GluN2A in LTD

As described above, the exact nature of the role of GluN2A in the LTP process is still not completely clear. Similarly, it is still not possible to make an educated guess on the exact role of GluN2A in LTD, as there are contradictory results on the matter. Some studies using the pharmacological approach revealed that a concentration of NVP-AAM077 that is able to impair LTP does not affect LTD in culture [129], in acute slices [110], or in vivo [130], suggesting that GluN2A is not necessary for LTD. However, there are other studies in opposition, in which the antagonist blocked LTD at the same concentration in which blocked LTP [112,113]. In another in vivo study, mice injected intraperitoneally with two different concentrations of NVP-AAM077 that were able to block LTP showed LTD impairments just at the higher concentration [114], suggesting that just a substantial reduction in NMDAR-mediated current and subsequent calcium entry is able to perturb LTD induction, whereas at low concentration of NVP-AAM077, the residual calcium influx is not able to trigger LTP but is still able to mediate LTD. Also, genetic approaches, providing mostly negative results, did not provide a convincing answer to the question. No LTD deficit was found in GluN2A knockout mice [131] and neither in case of direct [132] nor indirect GluN2A overexpression [133] at 1 Hz stimulation protocol, although at 3–5 Hz, GluN2A overexpression reduced LTD without affecting LTP [132]. It was demonstrated that NMDAR-mediated ion flux block could not stop LTD induction, suggesting the emerging idea that the basal state of the spine, rather than the amount of the calcium influx through NMDARs, is essential for LTD. However, to what extent GluN2A is involved in LTD, if through the intracellular association or the channel properties conferred to the NMDAR, it is still not determined, even if the data provided do not evidence a necessary role of the subunit in the process.

### 4.3. Role of GluN2A Interactors in Synaptic Plasticity

Despite GluN2A important role mediated by the particular channel properties it confers to the NMDAR, data seem to point out that many of the GluN2A specific effects, both in and out of the context of synaptic plasticity, may be due to the unique intracellular interactors that mediate GluN2A specific signaling pathways. First, as already described, some GluN2A CTD associated proteins are scaffolding proteins and mediate GluN2A membrane association and synaptic localization (See Figure 1). It was reported that Rph3A promotes stabilization of GluN2A-containing NMDARs in the postsynapse [71]. Franchini et al. recently published that Rph3A is targeted to the synapse after chemically induced LTP, which, in turn, drives the upregulation of GluN2A-containing NMDAR induced by LTP through the formation of the Rph3A-GluN2A-PSD-95 complex. Remarkably Rhp3A silencing in vitro blocks LTP induction. Interfering with Rph3A/GluN2A interaction produces the same effect and in vivo induces a strong memory deficit [125]. Other important interactors of GluN2A CTD that modulate its function in synaptic plasticity are protein kinases. Cdk5 can upregulate GluN2A function via phosphorylating GluN2A at Ser1232 in vitro and in vivo [87]. CaMKIIα competes with PSD-95 for binding to GluN2A [79]. This interaction occurs with non-phosphorylated CaMKII, but is positively regulated by kinase autophosphorylation [86]. CaMKII activity has been demonstrated to be necessary and sufficient for LTP induction (reviewed in [134]). Finally, GluN2A has the unique association with the synaptonuclear messenger RNF10 (see Figure 1) that has been demonstrated to mediate GluN2A-mediated LTP maintenance as well as LTP-dependent structural modifications of dendritic spines [76]. More recently Carrano et al. demonstrated a wider role of the GluN2A-associated synaptonuclear communication mediated by RNF10 in the regulation of dendritic arborization [135]. 

## 5. Role of GluN2A in Learning and Memory

Synaptic plasticity is considered the cellular and molecular basis for learning and memory formation [136,137] and several studies have demonstrated an important role for NMDARs in specific forms of learning and memory [138,139]. The specific involvement of GluN2A in learning and memory has been investigated through both pharmacological and genetical approaches. Genetic ablation of GluN2A and expression of a GluN2A lacking the CTD did not impair spatial tasks acquired over a number of days (the Morris water maze (MWM) and radial arm maze) [140]. Neto1 knockout mice, characterized by reduced level of GluN2A and LTP deficits, perform normally at MWM [118]. Pharmacological manipulations gave similar results: intraperitoneal injections of the GluN2A antagonist NVP-AAM077, able to block LTP, in vivo did not alter MWM acquisition or consolidation in rats [130]. However, interfering with GluN2A has been associated with short-term memory deficits. In fact, GluN2A-knockout or GluN2A C-terminus-deficient mice exhibit impaired spatial working memory [140]. Moreover, CA1 infusion of NVP-AAM077 impaired the performance on a delayed alternation T-maze task [141]. Other works suggested that GluN2A might participate in the rapid acquisition of context or object representations. Indeed, GluN2A knockout mice showed an impaired performance in a variant of a hippocampus-dependent contextual fear-conditioning with reduced context exposure before shock delivery [120]. Moreover, Neto1 knockout mice were impaired in a displaced object recognition task, but not in a novel object recognition task, suggesting a specific deficit in a hippocampal dependent test [118]. Similarly, Franchini et al. found that mice treated with a cell-permeable peptide that interferes with Rph3A-mediated GluN2A synaptic delivery also display memory defects in the object displacement task [125]. 

Thus, GluN2A does not seem necessary for incrementally acquired long-term memory tasks. It seems that GluN2A partially controls learning and memory: it is not necessary for long-term memory tasks but seems to affect short-term memory and the rapid acquisition of spatial information.

However, it is interesting to note that pharmacological and genetic manipulations of the GluN2B subunit produce similar cognitive alterations of the ones described by manipulating GluN2A, suggesting that a general reduction in NMDAR-mediated current might induce short-term memory deficits, rather than specifically interfering with GluN2A-containing NMDAR.

NMDAR has been subject of study also in the context of fear memory. Even if most of the studies support the idea that GluN2B is the subunit that is primarily involved in this kind of memory process [142,143,144,145,146], recently some studies have also pointed out an involvement of GluN2A-containing NMDARs. The group of Luo demonstrated that interfering with the activity-dependent insertion of GluN2A mediated by the ER chaperone Bip in the dorsal hippocampus was sufficient to impair the acquisition of fear conditioning test, suggesting that the interaction between the two proteins is crucial for fear memory formation [107]. Moreover, an increase of GluN2A/GluN2B ratio in basolateral amygdala after fear memory consolidation inhibits retrieval-dependent memory destabilization and modification of the fear memory trace, suggesting a neurobiological molecular explanation of why some memory events are resistant to variation and extinction [147]. Similarly to synaptic plasticity, the process of learning and memory also influences GluN2A. A hippocampal-dependent spatial memory task, MWM, was shown to upregulate GluN2A mRNA levels after the training session. Baez et al. showed an increase in GluN2A levels in the hippocampi in habituation of a new environment starting from 30 min after the test [126]. Interestingly, a similar upregulation of GluN2A was also found after each phase of object presentation of the novel object recognition task [24]. This evidence supports the idea that specific types of memory are supported by a contribution of GluN2A-containing NMDAR mediated neurotransmission and so the subunit is upregulated by the process itself. 

## 6. Role of GluN2A in Pathological Plasticity

Modifications of the GluN2A-containing NMDARs have been implicated in several pathological conditions including among others cerebral ischemia [148], depression [149,150,151,152,153,154,155,156], anxiety [149,157], schizophrenia [158,159,160,161], and Huntington’s disease [162,163,164]. In this review we decided to focus our attention on selected brain disorders in which GluN2A disfunctions is strictly correlated to altered synaptic plasticity, such as epilepsy, Alzheimer’s disease, Parkinson’s disease, Fragile-X Syndrome, and autism.

### 6.1. GluN2A in Epilepsy

Epilepsy represents a common disease in the population with complex pathophysiology leading to aberrant firing in brain circuits [165]. Several mutations of *GRIN2A* (encoding the GluN2A subunit) leading to NMDAR gain- or loss-of-function have been associated with different epilepsy-aphasia spectrum (EAS) such as benign epilepsy with centrotemporal spikes (BECTS), the Landau–Kleffner syndrome (LKS) and epileptic encephalopathy with continuous-spike-and-waves-during-slow-wave-sleep (CSWSS) [166,167,168]. A patient with the GluN2A P552R substitution presented seizures and intellectual disability [169]. This mutation affects the pre-M1 region of GluN2A leading to higher glutamate and glycine potency of the NMDAR, without impairments in receptor surface trafficking or expression. Hippocampal primary cultures transfected with this receptor variant showed increased neurotoxicity under synaptic stimulation conditions, which was rescued by memantine, underlining the contribution of this mutation to the clinical phenotype observed [170]. Other mutations associated with epilepsy, the GluN2A M817V as well as the L812M substitution which both reside in the pre-M4 region [171,172], increase agonist potency, channel mean open time and open probability while reducing endogenous negative modulator effects on receptor activity [173]. A GluN2A mutation at Tyr1387 was reported in a family displaying CSWSS and an autistic phenotype [167]. Interestingly, Tyr1387 is a well-known GluN2A phosphorylation site by Src Kinase, important for the modulation of the receptor activity [174]. 

Other *GRIN2A* mutations such as the GluN2A-I148S and R512H were associated with reduced surface NMDAR expression, activation time and current amplitude, when expressed in heterozygous or homozygous condition in HEK cells [175]. Overall, GluN2A-I148S and R512H can be considered dominant negative variants of GluN2A, as well as the I184T, C436R, R518H, T531M, V685G, and D731N [175,176,177,178]. Moreover, also the GluN2A-N651K variant previously described (see NMDAR structure paragraph) can be considered as a dominant negative form of the GluN2A subunit due to low Ca^2+^ influx in spite of low Mg^2+^ sensitivity [53]. Finally, there are some GluN2A mutations that are also present in GluN2B (equivalent sites) but give rise to different phenotypes. In particular, the GluN2A variants C436R, V685G, and D731N were shown to induce intractable epilepsy with developmental delay and general tonic-clonic seizures [167,176,177], whereas GluN2B ones (C436R, Q413G, and C461F) with intellectual disability and absence seizures [178,179,180].

However, the involvement of GluN2A subunit in epilepsy has been correlated not only to genetical variants, but also to altered trafficking, expression and balance with other NMDAR subunits for an adequate receptor functioning. 

Injection of convulsive agents, such as 3-mercaptopropionic acid or 4-aminopyridine for several days, is able to increase hippocampal GluN2A levels [181,182]. Chen and colleagues demonstrated how selective antagonism of the GluN2 subunits is neuroprotective in the hippocampus of pilocarpine and kindling animal models of epilepsy. However, only GluN2A antagonism reduced epileptogenesis, seizure-induced mossy fiber sprouting, and the activity-dependent BDNF expression indicating a crucial role of this subunit in epilepsy [183]. Recently, activation of GPR40 (a Gq coupled GPCR) in the kainic-acid (KA) induced epilepsy model and PTZ-kindling model reduced susceptibility to SE, most probably reducing NMDAR surface localization by regulating the endocytosis of the receptor [184]. 

A characteristic of some epilepsy models is that a single application of convulsive agent (electrical stimulation, GABA-A receptor antagonist, and Kainic Acid) has a long-term impact on electrical activity of the model itself, even after a washout period. Abegg and colleagues revealed how the Bicuculline (GABA-A receptor antagonist) epilepsy model induces abnormal LTP-like phenotype by exaggerated potentiation of excitatory synapses [185]. In particular, they observed how NMDAR activity was responsible for synapse potentiation by increasing synaptic AMPAR insertion, then occluding LTP induction but enhancing LTD amplitude in CA1. In this study, they provided a probable explanation for impaired learning abilities after epileptic exposure [186,187,188]. However, more recent literature identified both increased [189,190,191] and decreased LTP responses, probably depending on experimental conditions and how efficiently the seizure-induced LTP-like condition was realized [192,193,194]. Finally, in a recent work Hanson et al. found that the seizure phenotype was ameliorated by the use of a Positive Allosteric Modulator of GluN2A in a mouse model of Dravet syndrome [195]. In this case, the activation of GluN2A-containing NMDAR was able to improve the brain oscillations and neuronal hypersynchrony, improving the epileptic discharges. 

### 6.2. GluN2A in Autism Spectrum Disorder (ASD) and in Fragile X Syndrome (FXS)

Neurodevelopmental disorders characterized by reduced learning and social abilities are also called Intellectual Disabilities (IDs). Among IDs, ASD shows high variability in the severity of social-deficit interaction, communication, and stereotyped behaviors [196,197,198]. Genetic background as well as environmental exposure to different stressors play an impact on the disease emergence [199,200]. FXS is an X-linked disease characterized by IDs and often accompanied by autistic features. In particular, FXS is due to lack or altered expression in *FMR1* gene, encoding Fragile Mental Retardation Protein (FMRP), which binds mRNA [201] and regulates its transport and translation [202,203].

Among the several neuronal alterations detected in ASD and FXS, a convergent point is dysregulation in synaptic protein expression and activity, leading to altered intracellular signaling during neurotransmission. Interestingly, FMRP target mRNAs include both pre and postsynaptic proteins such as NMDARs, PSD-95, Shank1-3, Calcium channels, SNAP-25, and many others [204]. 

In particular, NMDAR and mGluR signaling modulating ERK/MAPK kinase activity have been associated with the pathophysiology of FXS [205]. Indeed, FXS-patient brain and Fmr1-KO mouse model show hyperactive ERK/MAPK pathway [206,207]. Furthermore, elevated activation of ERK was detected in Fmr1-KO mice and associated to overproduction of APPα derived from APP cleavage [208]. 

Synaptic plasticity events in different regions of Fmr1-KO animals are altered compared to WT. In particular, the LTP in CA1 region of hippocampus is impaired in animal models of FXS [209,210] as well as in other brain regions [211,212,213,214,215]. Importantly, Lundbye and colleagues recently reported the NMDAR-dependent LTP in CA1 of Fmr1-KO animals was restored to normal levels with selective inhibition of GluN2A subunit through different antagonists and negative allosteric modulators (NAM) or via GRIN2A deletion [216].

mGluR1/5-dependent LTD in CA1 was reported to be enhanced in Fmr1-KO animals compared to WT [216,217,218,219]. Interestingly, mGluR1/5 LTD was also recovered to basal levels via GluN2A inhibition [216]. 

A reduction of both LTP [217] and NMDAR-mediated LTD [220] were also recorded in perforant path-granule cells of Fmr1-KO animals. In agreement with the DG involvement in pattern separation aspects of learning [221], Fmr1-KO mice display no impairments in acquisition phase of Morris Water Maze [222,223], but have reduced context discrimination and altered memory extinction [222,224], which is thought to be dependent on NMDAR-LTD [225]. 

Recently, integrated transcriptome analysis revealed, among other genes, alterations of *GRIN2A* in the adult hippocampus of ASD mouse models [226], strengthening its role in the pathophysiology of neurodevelopmental disorders. 

### 6.3. Pathological Synaptic Plasticity in Parkinson’s Disease and Dystonia

A balanced cross-talk between dopaminergic and glutamatergic transmission in the striatum is essential for corticostriatal synaptic plasticity and motor activity [227]. In Parkinson’s disease, the degeneration of nigrostriatal dopaminergic neurons leads to complex alterations of the basal ganglia pathways involving also impaired synaptic plasticity of striatal spiny projecting neurons (SPNs) [228]. SPNs express GluN2A- and GluN2B-containing NMDARs [229,230] and an alteration of GluN2A/GluN2B subunit ratio of the striatal synaptic NMDAR is a key element in the regulation of motor behavior and synaptic plasticity in PD [231,232]. Interestingly, distinct levels of dopamine denervation differentially alter striatal synaptic plasticity and NMDA receptor subunit composition [231]. The complete depletion of striatal DA induces in the PD rat model by 6OHDA, mimicking advanced stages of the disease, results in the loss of both LTP and LTD and an increase of GluN2A/GluN2B ratio at synapses [230,231]. However, an early PD model characterized by only a partial DA depletion and mild motor symptoms shows a normal LTD and an altered LTP associated with a selective increased expression of synaptic GluN2A NMDARs. The use of cell permeable peptides able to modulate GluN2A interaction with scaffolding elements and, consequently, a physiological NMDAR subunit composition led to a full rescue of synaptic plasticity and to an amelioration of the motor behavior [231]. Overall, these studies indicate that an uncorrected composition of NMDARs is a key element in both motor behavior and synaptic plasticity in experimental PD. 

Notably, more recent studies suggest that also α-synuclein (α-syn) produces deficits in visuospatial learning and impairs LTP in striatal SPNs belonging to the direct and indirect basal ganglia pathways by altering the activity and synaptic localization of GluN2A-containing NMDARs [233]. In addition, α-syn impairs NMDAR-dependent LTP in striatal SPNs without inducing any effect on LTD [233], in agreement with previous reports showing that this latter form of synaptic plasticity in the striatum does not require NMDAR activation [228]. Similarly, also in hippocampal neurons α-syn oligomers impair LTP and modify basal synaptic transmission through an NMDAR-mediated mechanism [234]. Even if levodopa remains the major pharmacological approach for Parkinson’s disease, patients treated for several years develop disabling motor complications known as levodopa-induced dyskinesia [235]. These events are strictly correlated to adaptive changes of the glutamatergic corticostriatal signaling and involve also an aberrant functioning of NMDARs. Accordingly, the use of the low-affinity noncompetitive NMDAR antagonist amantadine allows at least a short-term benefit in the treatment of dyskinesia [235,236]. Of relevance, chronic levodopa treatment restores bidirectional synaptic plasticity (LTD, LTP, and depotentiation) only in non-dyskinetic conditions, whereas both in animal models and in patients, dyskinesias are associated with the loss of depotentiation of previously induced LTP [210,237,238]. 

Several reports demonstrated an increased synaptic GluN2A/GluN2B ratio of NMDARs in dendritic spines of SPNs, both in animal models and in patients [230,232,239], paralleled by modifications in the association of GluN2-type subunits with scaffolding proteins. Interestingly, treatment with peptides disrupting GluN2A interaction with MAGUK proteins or with Rph3A demonstrated that a decrease in synaptic GluN2A-containing NMDAR is sufficient to induce a significant reduction in the severity of Levodopa-induced dyskinesia [240,241]. 

Alterations of synaptic plasticity have been reported as a key determinant in the pathophysiology of early-onset generalized torsion dystonia (DYT1), an autosomal dominant movement disorder [242]. Human studies identified modifications of neuronal processing and plasticity in dystonic patients [243] and an impairment of striatal plasticity has been shown in different DYT1 animal models. In particular, a recent study demonstrated in a DYT1 dystonia mouse model that LTP appeared prematurely in a critical developmental window in striatal SPNs, whereas LTD was never recorded [244]. Notably these functional alterations where closely related to an increase of postsynaptic GluN2A suggesting a “premature” GluN2A/GluN2B switch [244].

### 6.4. Dysfunction of Glutamatergic Synaptic Plasticity in Alzheimer’s Disease

Dysfunction of the excitatory synapse and spine loss represent early functional and morphological events of Alzheimer’s disease (AD). Several postsynaptic proteins of the glutamatergic synapses have been demonstrated to be a key target of the amyloid-β_1–42_ (Aβ_1–42_) peptide, whose accumulation and deposition are the main hallmarks of AD. Notably, several studies indicate that Aβ_1–42_ is an important modulator of neuronal activity affecting in turn synaptic transmission and plasticity [245]. In particular, oligomeric Aβ_1–42_ can alter the activation of NMDARs and downstream signaling cascades promoting the loss of LTP, the induction of LTD, and synaptic loss [246,247,248]. 

The different contribution of GluN2A- and GluN2B-containing NMDARs to Aβ_1–42_-induced synaptic toxicity has been widely evaluated. Several studies reported the role of the extra-synaptic GluN2B-containing NMDARs in these events [249,250,251]. Aβ_1–42_ oligomers induce intracellular Ca^2+^ overload particularly mediated by the GluN2A subunit and neuronal death that can be prevented by NMDAR antagonists [252]. In addition, another study showed that presenilin knockout mice models are characterized by an early increase in GluN2A-containing NMDARs at the PSD with a concomitant reduction at non-synaptic sites before synaptic loss [253]. Moreover, oligomeric Aβ_1–42_ caused selective loss of synaptic GluN2B responses, promoting a switch in subunit composition from GluN2B to GluN2A, a process normally observed during development [254]. As GluN2A subunits have been implicated in protective pathways, whereas GluN2B subunits appear to increase neuronal vulnerability [255], the early increase in GluN2A and decrease in GluN2B subunit-composed NMDARs activity may be an attempt to reduce Aβ_1–42_-induced neuronal dysfunction. Finally, a very recent study provided a novel mechanistic explanation for Aβ_1–42_-dependent aberrant plasticity induced oligomeric forms [256]. In particular, Marcello and co-workers demonstrated that oligomeric Aβ_1–42_ activates a pathway that requires neuronal activity and the involvement of the GluN2A-containing NMDARs. Moreover, Aβ_1–42_-induced neuronal degeneration involves activation of extrasynaptic GluN2B-containing NMDARs followed by increased Tau phosphorylation, whereas spine loss can be mainly mediated by GluN2A-containing NMDARs signaling and active caspase-3 [257]. Noteworthy, among Aβ toxicity mechanisms, also NMDAR metabotropic signaling was shown to impact on synaptic functions. However, this process seems to mainly involve GluN2B-containing NMDARs as reported by Malinow’s laboratory in 2013 [254]. However, Aβ treatment was also found to act indirectly on GluN2A subunit, increasing the synaptic GluN2A/GluN2B ratio [254] and so inducing a switch in NMDAR subunits similar to the neurodevelopmental one.

The impact of GluN2A subunit in AD has been recently taken into account also as a potential pharmacological target. In particular, GNE-0723, a novel positive allosteric modulator of GluN2A-containing NMDAR, was shown to ameliorate the cognitive deficits in a mouse model of AD (J20) [195], supporting the importance of the involvement of synaptic NMDAR neurotransmission in AD pathogenesis. 

## 7. Conclusions

NMDARs have been intensively studied in the last decades because of their involvement in many aspects of neuronal transmission as well as learning and memory. It has been clearly demonstrated that NMDAR properties depend on its subunit composition and this review focused on the distinctive functions of synaptic GluN2A-containing NMDARs. Even if these NMDAR subtypes play a key role in both physiology and pathological synaptic plasticity, future studies aiming to understand their contribution to the pathophysiology of neurological disorders will require novel and specific approaches to overcome the limitations due to the high similarity among GluN2-type regulatory subunits. At present, genetic manipulation of GluN2A did not fully clarify its pathophysiological role and the available pharmacological tools are not specific enough to give a better answer. Understanding the role and the modulation of GluN2A protein–protein interactions at the CTD may provide new possible targets for pharmacological tools and be a future direction in which to investigate the unique role of this NMDAR subunit in the context of glutamatergic neurotransmission and synaptic plasticity.
